# Sperm Repository for a Breeding Program of the Eastern Oyster *Crassostrea virginica*: Sample Collection, Processing, Cryopreservation, and Data Management Plan

**DOI:** 10.3390/ani11102836

**Published:** 2021-09-28

**Authors:** Huiping Yang, Yuanzi Huo, Jayme C. Yee, Scott Rikard, William C. Walton, Eric Saillant

**Affiliations:** 1 Institute of Food and Agricultural Sciences, School of Forest, Fisheries and Geomatics Sciences, University of Florida, Gainesville, FL 32653, USA; yuanzihuo@ufl.edu (Y.H.); jayme.yee@ufl.edu (J.C.Y.); 2 Auburn University Shellfish Laboratory, School of Fisheries, Aquaculture and Aquatic Sciences, Auburn University, Dauphin Island, AL 36849, USA; rikarfs@auburn.edu (S.R.); walton@vims.edu (W.C.W.); 3 Virginia Institute of Marine Science, College of William & Mary, Gloucester Point, VA 23062, USA; 4 Thad Cochran Marine Aquaculture Center, School of Ocean Sciences and Technology, University of Southern Mississippi, Ocean Springs, MS 39564, USA; Eric.Saillant@usm.edu

**Keywords:** sperm repository, germplasm, cryopreservation, breeding, eastern oysters *Crassostrea virginica*, aquaculture, Gulf of Mexico

## Abstract

**Simple Summary:**

The Eastern oyster *Crassostrea virginica* is one of the most important fishery and aquaculture species in the USA and is a keystone species for coastal reefs. A breeding program was initiated in 2019 to support the fast-growing aquaculture industry for this species in the Gulf of Mexico. Oysters from wild populations in embayment along the U.S. Gulf of Mexico coast were used as broodstock for the program to maximize genetic diversity. A sperm repository of the broodstock, including a total of 102 male oysters from the 17 collection sites, was established to support the breeding project. Sperm collection was accomplished by strip spawn, and fresh sperm production, motility, and fertility were recorded for quality analysis. Cryopreserved sperm samples were sorted, labelled, archived, and stored in liquid nitrogen for future use. Post-thaw motility and plasm membrane integrity were recorded as post-thaw quality parameters. Overall, this study demonstrated sperm sample collection, processing, cryopreservation, and a data management plan involved in the establishment of the sperm repository. The streamlined procedure can serve as a template for construction of oyster germplasm repositories for breeding programs.

**Abstract:**

The Eastern oyster *Crassostrea virginica* (Family Ostreidae) is one of the most important fishery and aquaculture species in the U.S. and is a keystone species for coastal reefs. A breeding program was initiated in 2019 to support the fast-growing aquaculture industry culturing this species in the Gulf of Mexico. Oysters from 17 wild populations in embayment along the U.S. Gulf of Mexico coast from southwest Florida to the Matagorda Bay, Texas were used as broodstock for the program to maximize genetic diversity in the base population. A sperm repository of the broodstock was established to support the breeding project. The goal of this study was to demonstrate the sperm sample collection, processing, cryopreservation, and the data management plan involved in the establishment of a sperm germplasm repository of base populations. The supporting objectives were to: (1) develop a data management plan for the sperm repository; (2) streamline the procedure for sample collection, processing, and cryopreservation; (3) incorporate sperm quality analysis into the procedure, and (4) archive the cryopreserved samples as a repository for future use in the breeding program. This sperm repository included a total of 102 male oysters from the 17 collection sites (six oysters per site). A data management plan was developed with six categories, including sample collection, phenotype, fresh sperm, genotype, cryopreservation, and post-thaw sperm, as guide for data collection. Sperm collection was accomplished by strip spawn, and fresh sperm production, motility, and fertility were recorded for quality analysis. Cryopreserved sperm samples were sorted, labelled, archived, and stored in liquid nitrogen for future use. Post-thaw motility (1–30%) and plasm membrane integrity (15.34–70.36%) were recorded as post-thaw quality parameters. Overall, this study demonstrated a streamlined procedure of oyster sperm collection, processing, and cryopreservation for establishing a sperm repository that can serve as a template for construction of oyster germplasm repositories for breeding programs.

## 1. Introduction

The Eastern oyster *Crassostrea virginica* (Family Ostreidae) is one of the most important fishery and aquaculture species in the U.S. and is a keystone species for coastal reef and ecosystem services [1]. The Eastern oyster is distributed naturally in eastern North and South America ranging from northern New Brunswick through parts of the West Indies and south to Brazil and the Gulf of Mexico [2]. The harvest of eastern oysters as food by hand or tongs dates back to at least the 17th and 18th centuries [3]. In the early 1800s, dredging of oysters grew quickly. This technique was a major contributor to the decline of stocks by allowing harvesting oysters in areas that could not be accessed with other methods. In the late 1950s, oyster fisheries in the Chesapeake and Delaware Bays collapsed. The decline in abundance was largely attributed to MSX (*Haplosporidium nelsoni*) and Dermo (*Perkinsus marinus*) diseases [3,4]. The Gulf of Mexico fishery is the largest contributor to U.S. oyster production. The decline of wild harvests in that region took a precipitous turn in 2012, when a sharp decrease occurred due, at least in part, to a prolonged drought [5]. In 2020, oyster harvesting in the iconic Apalachicola Bay, Florida, U.S. was shut down in December for at least five years because of the high level of depletion of wild oyster beds [6].

Modern oyster aquaculture was initiated in the 1920s and became established in the early 1960s when larval culture methodologies were developed [7] and microalgae, an essential food for oyster larvae, could be cultured at a large scale to supply the needs of hatcheries [8,9]. With the decline of oyster fisheries in the coastal U.S., aquaculture production has grown rapidly since the 1960s [10]. Genetic improvement largely aimed to address disease mortality by producing resistant lines [11]. For example, six MSX-resistant strains have been bred at Rutgers University since the 1960s and two other strains Delaware Bay and Northeast High Survival lines (DBH and NEH) were subsequently produced by crossing previously developed resistant lines [12]; dual disease-resistant strains to MSX and Dermo were also produced [13], and the largest oyster-breeding program at the Virginia Institute of Marine Sciences (VIMS) (http://www.vims.edu/research/units/centerspartners/abc/index.php, 24 September 2021) started implementing a family selection program in the mid-2000s, which is now incorporating genomic selection. These programs have been supporting a major part of the U.S. East Coast oyster aquaculture industry.

Cryopreservation is a technology used to freeze biological materials to ultra-low temperatures (usually at −196 °C in liquid nitrogen). Germplasm cryopreservation is an essential tool for breeding programs and has been widely employed in plant [14] and livestock [15] breeding. The applications include preservation of base populations to maintain genetic diversity, preservation of each breeding generation to allow strategic breeding (e.g., backcross), and long-term preservation of stabilized superior strains for commercial use. Research on germplasm cryopreservation in mollusks was first reported in sperm of the Pacific oyster *Crassostrea gigas* in 1971 [16]. To date, over 80 reports have been published on molluscan germplasm cryopreservation [17], and the studied species were all aquaculture species, primarily oysters [18], but also mussels, scallops, clams, and abalones [17,19]. The targeted germplasm for cryopreservation included sperm [19], oocytes, embryos, and larvae [20]. 

For Eastern oysters, germplasm cryopreservation has been studied in sperm [21,22,23,24] and larvae [23,25] (Table 1). One laboratory protocol was developed by the author of the current study through systematic evaluation of cryoprotectants, cooling rates, and thawing temperatures [24], and has been used to produce inbred lines through fertilizing cryopreserved sperm with oocytes from the same individual oysters after sex reversal [26]. In the current study, this protocol was employed to establish a sperm repository of a base population of *C. virginica* for a breeding project.

A germplasm repository requires representation and diversity. Representation of a germplasm includes viability, quantity, and coverage of a species, population, landrace, hybrid, or cultivar. Genetic diversity metrics, such as allele frequencies, gene diversity indices, heterozygosity and number of alleles, and populations origins need to be considered to determine a sampling strategy [27]. For breeding programs, the actual contribution of available individuals to a generation, measured as the effective number of breeders, is the relevant parameter that needs to be maximized to avoid bottlenecks which constrain genetic variability [28]. Genetic markers have been widely used to assess the contribution of germplasm samples in conservation activities and use of plant germplasm. The diversity of a germplasm repository for a breeding program is usually defined by its strategic breeding plan.

To support the fast-growing oyster aquaculture industry in the Gulf of Mexico, a selected breeding program was initiated in 2019. This breeding project aims to develop Eastern oysters with superior genetic values for traits critical to the industry based on Gulf genotypes in a family selection approach. A repository of genetic resources from regional populations used as broodstock (or base populations) and selected lines adapted to environmental conditions will be established to support the breeding project and future oyster restoration efforts.

The goal of this study was to demonstrate the sperm sample collection, processing, cryopreservation, and the data management plan involved in the establishment of a sperm germplasm repository of base populations. The supporting objectives were to: (1) develop a data management plan for the sperm repository; (2) streamline the procedure for sample collection, processing, and cryopreservation; (3) incorporate sperm quality analysis into the procedure, and (4) archive the cryopreserved samples as a repository for future use in the breeding program. It is expected that this report may serve as an example or template for establishing germplasm repositories for breeding programs.

## 2. Materials and Methods

### 2.1. Broodstock Collection

**Figure 1:**
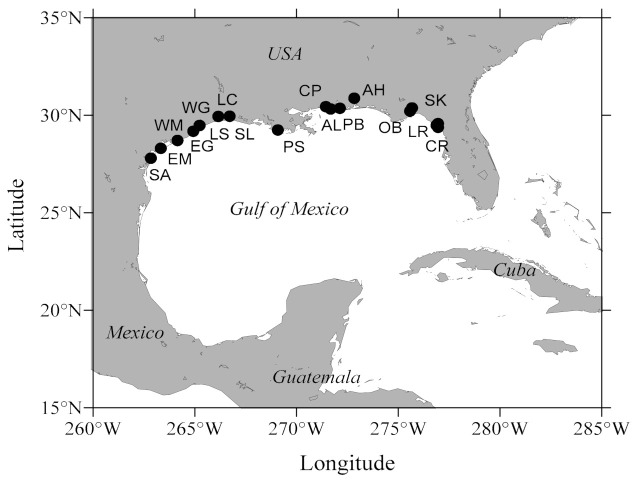
Schematic map showing Eastern oyster *Crassostrea virginica* 17 collection sites (in order of decreasing longitude) along the Gulf of Mexico). **Florida**: Corrigan Reef (CR), Lone Cabbage Reef (LR), Seahorse Key (SK), Oyster Bay (OB), Alligator Harbor (AH) and Pensacola Bay (PB). **Alabama**: Alonzo Landing (AL) and Cedar Point (CP). **Mississippi:** Pascagoula (PS). **Louisiana**: Sister Lake (SL) and Lake Calcasieu (LC). **Texas**: Lake Sabine (LS), West Galveston Bay (WG), East Galveston Bay (EG), West Matagorda Bay (WM), East Matagorda Bay (EM) and San Antonio Bay (SA).

Adult Eastern oyster *Crassostrea virginica* broodstock (*n* = 100–200) were collected from 17 locations along the Gulf of Mexico (Figure 1) from Florida to Texas in spring and summer 2020. After collection, broodstock were transported to the Auburn University Shellfish Laboratory (AUSL) where they were held through summer in cages at the AUSL field site (Grand Bay, AL, USA) to allow reproductive conditioning for a spawning event planned in late August. No water quality data were recorded.

### 2.2. Breeding Strategy

Spawning and breeding were performed at AUSL from 31 August to 3 September 2020. A total of 102 male oysters were processed for sperm collection with six males from each collection site. Two hundred and four full-sib families were produced according to 51 of 2 × 2 non-overlapping factorial mating sets. Sperm from each male fertilized oocytes from two females from different collection sites, and oocytes from each female were fertilized with sperm from two males to achieve the design matrix. After collection, sperm samples were used for fertilization and surplus sperm was cryopreserved for the repository.

### 2.3. Sperm Collection

**Figure 2:**
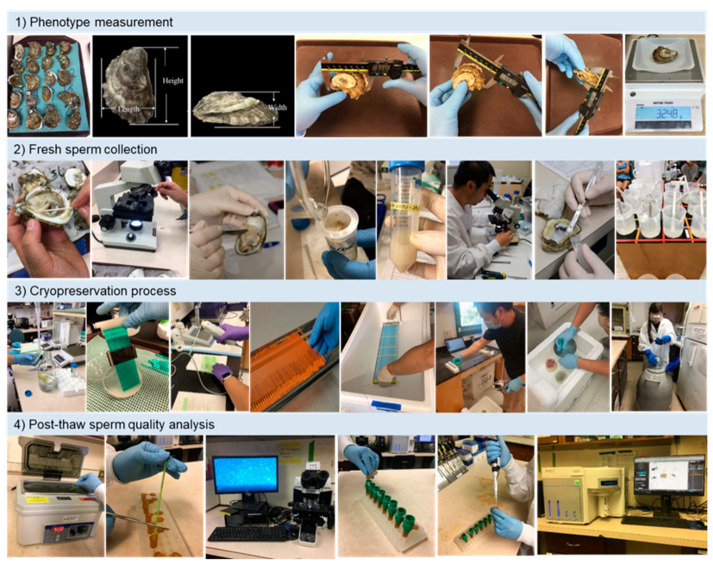
The procedure of sperm collection, processing, cryopreservation, and post-thaw quality analysis. (1) **Phenotype measurement** (from left to right): lining oysters, height and length standard, width standard, height, length, width, and whole-body weight. (2) **Fresh sperm collection**: sampling a piece of gonad, observation for sex determination, dissection of testis, filtering sperm suspension, sperm suspension in 50 mL tubes, determination of sperm concentration and motility, sampling of adduct muscle for genotype, fertility test of fresh sperm. (3) **Cryopreservation process**: mixing with cryomedium, packaging, sealing, arranging on freezing rack, loading into freezer, removing samples to liquid nitrogen after cooling to −80 °C, sorting samples, and storage in dewars. (4) **Post-thaw sperm analysis**: thawing sample straw, releasing sample into a 1.5 mL tube, post-thaw motility, dilution of post-thaw sample (100×), filter through 20 µm screen, staining with SYBR-14/propidium iodide for membrane integrity analysis, and analysis using flow cytometer.

Oysters from a given collection site were randomly lined up in a tray and marked with a permanent marker. Shell height, length, and width were recorded (Figure 2) using a digital caliper (0.01 mm accuracy, Mitutoyo, Aurora, IL, USA), and the total body weight of individuals was recorded using an electronic scale (0.0001 g accuracy, Mettler Toledo ME4002E).

After measurements, each oyster was opened, and the upper shell was removed carefully with a sterilized oyster knife to avoid contamination. Based on visual observation of gonad development, oysters with better gonad development were kept for spawning. A piece of gonad was sampled from each oyster and viewed by use of a compound microscope at 100× magnification (Olympus, BX43, Tokyo, Japan), and sex was determined by the presence of oocytes or sperm.

Testis development from each male oyster was photographed before dissecting. Testis from each male was carefully stripped into a pre-weighed 100 mL beaker using a sterilized scalpel, and testis weight was recorded. Sperm were released by crushing the testis into Ca-free HBSS at an osmolality of 650 mOsmol/kg (Ca-free HBSS650) [29] at a ratio of 5 mL of HBSS per 1 g testis. Sperm suspensions were filtered through a 70-μm Nitex screen to remove debris into a 50 mL centrifugation tubes, and sperm volume was recorded. Ca-free HBSS650 was prepared by adjusting the water volume from the standard recipe of HBSS 1 L to around 450 mL without the addition of CaCl2 (0.137 M NaCl, 5.4 mM KCl, 1.0 mM MgSO4, 0.25 mM Na2HPO4, 0.44 mM KH2PO4, 4.2 mM NaHCO3, and 5.55 mM glucose, pH = 7.8) [29].

After sperm collection, one piece of adductor muscle (~1 cm^3^) was sampled, cut into small pieces using a sterilized scalpel, and transferred into 95% ethanol in 5 mL centrifuged tubes for DNA extraction and genotyping. The ethanol was replaced once at the end of the sample day, and samples were stored at −20 °C until processing.

### 2.4. Determination of Sperm Concentration

Sperm concentration was determined by use of a hemocytometer (Bright-Line™ Counting Chamber, Fisher Scientific). Specifically, the sperm sample was diluted 100 times (2 µL sperm in 190 µL fresh seawater plus 8 µL ethanol). After mixing well, a 10 µL sample was loaded on the hemocytometer, and sperm concentration was counted at a 200× magnification using a microscope. The original sperm concentration was calculated and recorded.

### 2.5. Estimation of Fresh Sperm Motility and Fertility

Sperm from Eastern oysters (and most molluscan bivalves) begin to swim (become activated) when suspended in sea water or buffers at suitable osmolarities and can swim continuously for as long as 5 h [2]. In this study, sperm motility was estimated using visual observation using a microscope at 200× magnification within 1 h after suspending in Ca-free HBSS650. Specifically, 1 µL of sperm suspension was sampled on a slide, and sperm motility was observed and recorded immediately following the addition of 9 µL fresh seawater (650 mOsmol/kg) to dilute the sperm (Figure 2).

Fertilization was conducted by gently mixing sperm and oocytes to achieve a ratio of 10 spermatozoa per oocyte (the ratio was monitored by observing spermatozoa surrounding oocytes under a microscope at 100× magnification). Oocyte collection was performed by stripping gonads. Gonads were stripped by gently scrapping oocytes with a scalpel directly in filtered seawater (T = 26–28 °C). The oocyte suspension was filtered through a 250 µm screen to remove large pieces of ovarian tissues, and oocytes were collected on a 20 µm screen and washed into a 4 L beaker in a 500 mL volume, where they were held for a 30 min hydration period. After mixing sperm and oocytes, the fertilized eggs were sampled every 2–5 min and observed using microscopic at 100× magnification until the first polar body in fertilized eggs was observed (10–20 min after mixing sperm and oocytes). Fresh seawater was then added to a volume of 4 L to reduce the density of fertilized eggs and ensure water quality remained adequate throughout embryo development. At the two-cell stage (about 1 h post-fertilization) or beyond, the embryo suspension (1 mL) was sampled after mixing, and the fertilized egg (with two or more embryonic cells visible) and total eggs were counted by use of a 1 mL Sedgewick Rafter counting chamber. The fertilization rate was calculated as the percentage of fertilized eggs from the total eggs. The same number of fertilized eggs from each family was combined into one group from each day of crosses for ‘common garden’ culture.

### 2.6. Sperm Cryopreservation Process

After fertilization was accomplished, the surplus sperm suspension from each male was processed for cryopreservation (Figure 2). Based on the surplus sperm volume and concentration, sperm concentration was adjusted to 1 × 10 ^9^ cells/mL for cryopreservation [24]. If the concentration was below 1 × 10 ^9^ cells/mL, no sperm concentration adjustment was needed.

Sperm cryopreservation was performed by following the protocol established in our previous research [24]. In a single step, sperm suspensions were mixed with the same volume of pre-made 20% dimethyl sulfoxide (DMSO) in Ca-free HBSS650 (yielding a final concentration of 10%), and the mixture was packaged into pre-labelled 0.5 mL straw using a filling station (IMV Technologies, Maple Grove, MN, USA). Straws were sealed by use of an ultrasonic sealer (Ultra-seal 21, Minitube, Verona, WI, USA) or an impulse heat sealer (AIE-105T, American International Electric, Inc., City of Industry, CA, USA). After a 20 min equilibration at room temperature, the sample straws were cooled by use of an aeration freezing system developed for field use (Huo et al., in review) at a cooling rate of 10–15 °C/min to reach −80 °C (temperature was monitored during cooling using a probe inserted within one straw alongside the sample straws). When samples reached −80 °C, the frozen samples were removed from the freezer, plunged into liquid nitrogen, and sorted into Daisy goblets (IMV Technologies, Maple Grove, MN, USA) for long-term storage in a liquid nitrogen Dewar.

### 2.7. Post-Thaw Sperm Quality Analysis

Post-thaw sperm viability, including motility and membrane integrity, were estimated after 3 months of storage in liquid nitrogen (Figure 2). Following the protocol [24], sample straws were removed from liquid nitrogen and immediately submerged into a water bath at 50 °C for 6 s. Thawed sperm straws were released into separate 1.5 mL centrifuge tubes on ice by cutting one end of the straw after wiping the straws dry with tissue paper. Post-thaw sperm motility was estimated by visual observation by use of a microscope (Olympus, BX43, Tokyo, Japan), as described above for fresh sperm assessment.

Plasma membrane integrity was analyzed as a parameter of sperm quality. The LIVE/DEAD^®^ SYBR-14/propidium iodide (PI) assay kit (Invitrogen, ThermoFisher Scientific, Eugene, OR, USA) was used by following the manufacturer’s instructions. Immediately after thawing, post-thaw sperm samples were diluted 100 times (10 µL post-thaw sperm plus 990 µL HBSS650, yielding a concentration of 1–5 × 10^6^ cells/mL) and filtered through a 20 µm screen. A 500 µL sample was stained with 100 nM SYBR-14 and 12 µM PI for 10 min in the dark and analyzed using a flow cytometer (Attune™, Thermo Fisher Scientific, Eugene, OR, USA) equipped with 488 nm excitation lasers. Before analyzing samples, the flow cytometer was tested by using fluorescent validation beads to ensure all quality parameters were passed. Events from a 50 µL sample were collected at a flow rate of 25 µL /min.

Flow cytometry data were analyzed using the manufacturer-provided software (Attune™ NxT Software). The sperm population was gated to exclude additional cell debris based on plots displaying forward scatter (FSC) vs. side scatter (SSC). Post-thaw sperm concentration was recorded using the gated total cell number and sample volume recorded by the flow cytometer after conversion to account for the dilution factor. Gated cells were analyzed on a scatter plot of BL1 (SYBR 14) vs. BL3 (PI) with fluorescence compensation (BL1 was compensated by BL3 with 0%, and BL3 was compensated by BL1 with 7.89%) to reduce spectral overlap. The sperm cells with intact plasma membranes were stained with SYBR-14, whereas cells stained with PI had damaged plasma membranes. Membrane integrity was expressed as the percentage of cells stained with SYBR-14 over the total cells stained with SYBR-14 and PI.

### 2.8. Data Analysis

Data collection, including shell metrics, whole body weight, sperm production, fertilization rate, motility, and membrane integrity were expressed as mean ± standard deviation. Data analysis was performed by JMP pro software (version 15.0, SAS Institute, Cary, NC, USA). Tests of homogeneity of variance were conducted and percentage data were arsine-transformed for normalization before analysis. ANOVA and correlations were used for data analyses. Tukey’s least significant difference was used to make post hoc comparisons between different combinations when significant effects were found. Differences were considered significant at *p* < 0.050.

The authors confirm that the U.S. National Research Council’s guidelines for the Care and Use of Laboratory Animals were followed. No IACUC-approved protocol was required for invertebrates.

## 3. Results

### 3.1. Data Management Plan in the Sperm Repository of Base Populations

**Figure 3:**
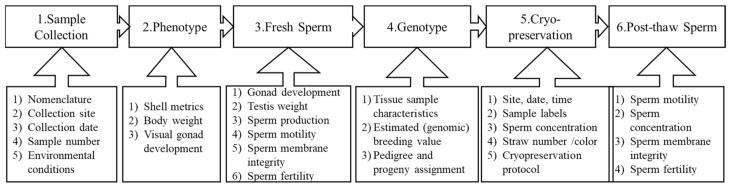
Schematic procedure for Eastern oyster *Crassostrea virginica* sperm collection and processing for breeding and cryopreservation. Parameters recorded during the process are reported below each individual step.

The purpose of this sperm repository was to preserve the base population for an oyster breeding program. A strategic data management plan was developed for this repository. The information recorded was grouped into six categories (Figure 3). For each category, data parameters recorded during collection were as follows.

(1) Sample collection metadata

Sample collection information recorded included the following: collection site geographic information (latitude, longitude, and name); collection date (year, month, day); total oyster number obtained at the location during the collection event; basic environmental conditions (temperature, salinity, pH values, oxygen concentration); oyster source (wild or farmed, aquaculture method such as bottom cages, floating cages, floating bags, or long-line cages); and collectors’ names. The information of each male and female used in this breeding program was determined and recorded in the program database before spawning.

(2) Phenotypic characteristics

Phenotypic characteristics of each oyster were recorded in the sperm repository, including shell metrics (height, length, and width) and body weight. The gonad development of each male oyster was photographed and attached to each individual oyster as one qualitative phenotypic parameter.

(3) Fresh sperm information

Parameters for fresh sperm information (quantity and quality) included testis weights, and gonad index (description of developmental condition), total sperm production, fresh sperm motility, and fresh sperm fertility indicated by “fertilization rate”. If the condition permits, the fresh sperm membrane integrity should be measured and recorded.

(4) Linking to genetic and genomic information

Tissue samples for genetic analysis taken at the time of sperm collection from each male and female brooder will be used for future genotypic analysis. A unique identification system was used to label straws and genetic samples ensured linking of sperm samples with genetic data when they were available.

(5) Sperm cryopreservation

Parameters for sperm cryopreservation of each male oyster included the working site, date, sample labels (matching with the oyster nomenclature), sperm concentration, quantity, straw number, color, cryopreservation protocol (including cryoprotectants, equilibration time, cooling rates), storage location (including goblet name, location, Dewar number), and sample inventory.

(6) Post-thaw sperm information

Post-thaw parameters included thawing temperature, post-thaw amendment strategy, post-thaw sperm motility, membrane integrity, sperm concentration, and sperm fertility. If possible, sperm fertility needs to be confirmed as a post-thaw sperm quality parameter.

### 3.2. Phenotypic Characteristics

A total of 102 male oysters (17 sites with 6 males from each site) were included in this sperm repository. The shell metrics and body weight (mean ± SD) from six male oysters collected from each site were calculated and listed in Table 1. Overall, these male oysters had an average shell height of 90.11 ± 7.29 mm ranging from 80.33 to 111.87 mm, shell length of 57.80 ± 7.57 mm ranging from 46.57 to 73.40 mm, and shell width of 34.16 ± 6.31 mm ranging from 25.36 to 43.30 mm. The overall average body weight was 141.21 ± 64.46 g ranging from 72.66 to 280.50 g (Table 2). All oysters used were larger than the market size (76.2 mm, 3 inches of height).

### 3.3. Fresh Sperm Production and Motility

**Figure 4:**
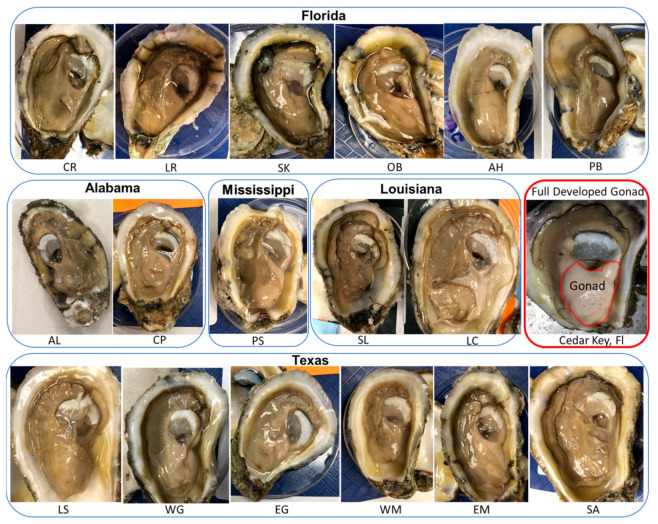
Photographs of gonad development condition of male Eastern oysters *Crassostrea virginica* included in the sperm repository. One male is presented out of the six males from each of the 17 collection sites along the Gulf of Mexico coast. One example of a fully developed gonad condition of an Eastern oyster (collected from Cedar Key in Florida in April 2021) is present here as a comparison.

Based on visual observation, male oysters were overall in poor gonad development condition (Figure 4). The gonad development condition is shown with one photograph from each collection site. Overall, testis weight (mean ± SD, *n* = 102 male oysters) was 2.08 ± 0.71 g ranging from 1.19 to 3.54 g. Gonadosomatic index (percentage of testis weight out of the total body weight) was 1.77 ± 0.95% ranging from 0.50% to 3.5%.

Sperm concentration averaged 0.68 ± 0.52 × 10^9^ cells/mL and ranged from 0.09 × 10^9^ cell/mL to 2.01 × 10^9^ cells/mL. Total sperm production averaged 1.05 ± 0.89 × 10^10^ cells and ranged from 0.10 × 10^10^ cells in oysters collected from CR to 3.11 × 10^10^ cells in oysters collected from CP (Table 2). Sperm motility averaged 38 ± 20% and ranged from 11 ± 8% in oysters collected from CR to 75 ± 5% in those collected from EG (Table 2).

### 3.4. Fertility Test of Fresh Sperm

**Figure 5:**
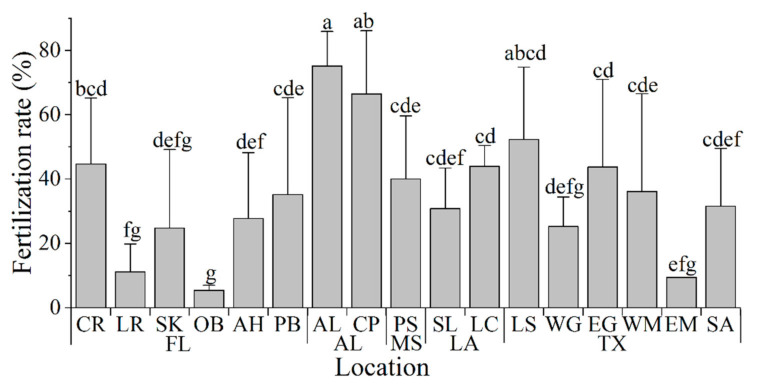
Fertilization rate (%) observed using fresh sperm of Eastern oysters *Crassostrea virginica* (*n* = 6, each male was used to cross two females) from different collection sites in the Gulf of Mexico. Groups labeled with the same letters (a–f) are not statistically different (*p* > 0.050).

Fresh sperm from each male was used to fertilize oocytes from two females. The fertilization rates observed for the fertilizations performed with each of the six males used from each collection site were calculated. Overall, the fertilization rates varied significantly among sample collection sites (Figure 5), averaging 34 ± 18% across all locations, and ranged from 5% to 75% for individual locations. The highest fertilization rates (75 ± 11% and 66 ± 20%) were obtained using sperm from oysters sampled at AL and CP (Figure 5). The lowest average fertilization rates (from 5% to 25%) were observed during fertilizations using sperm from oyster samples collected at SK, LR, OB, WG, and EM (Figure 5).

### 3.5. Post-Thaw Sperm Viabilities

**Figure 6:**
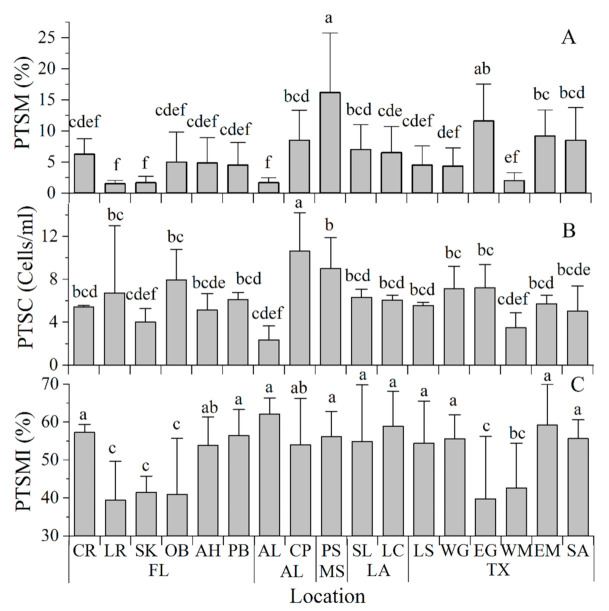
Post-thaw sperm quality analyses in Eastern oysters *Crassostrea virginica* from 17 collection sites along the Gulf of Mexico. (**A**). Post-thaw sperm motility (PTSM, %); (**B**). Post-thaw sperm concentration (PTSC, cells/mL), and (**C**). Post-thaw sperm membrane integrity (PTSMI, %). Groups labeled with the same letters (a–f) are not statistically different (*p* > 0.05).

The post-thaw sperm motility (PTSM) ranged from 2% to 16% with an average of 6 ± 4% across collection locations. The post-thaw sperm motility of male oysters varied greatly between collection sites. It averaged 16 ± 10% for oysters collected at PS and 12 ± 6% for those obtained at EG. The PS group had significantly higher PTSM values than those recorded at other locations (Figure 6A). PTSMs for oysters collected at the SK, LR, OB, AH, CR, PB, AL, WG, WM, and LS locations ranged from 2–6% and were significantly lower than those at other locations. Based on our experience with cryopreserved oyster sperm, the PTSM is typically in the range of 20–50% by visual observation. The low post-thaw sperm motility in this study likely reflected the poor gonad development condition at the time of strip-spawning.

The post-thaw sperm concentration ranged from 2.35 ± 1.29 × 10^8^ cells/mL to 10.61 ± 3.59 ×10^8^ cells/mL and averaged 6.10 ± 1.98 × 10^8^ cells/mL across all collection locations. After thawing, the sperm concentration varied greatly between locations. The post-thaw sperm concentration was on average 10.61 ± 3.59 × 10^8^ cells/mL at CP, which was higher than those at other collection sites (Figure 6B).

The post-thaw sperm membrane integrity ranged between 39 and 62% with an average of 52 ± 8% across all collection locations. The post-thaw sperm membrane integrity ranged from 39 ± 10% to 42 ± 4% at the SK, LR, OB, EG, and WM locations. These values were significantly lower than those at CR, PB, AL, PS, EM, WG, SA, LS, SL, and LC which ranged from 54 ± 11% to 62 ± 4% (Figure 6C).

### 3.6. Correlation between Parameters

Significant positive correlations were found among shell metrics and body weight (*p* < 0.001) (Table 3). However, testis weight was not correlated with the shell metrics (*p* ≥ 0.323) or body weight (*p* = 0.187) (Table 3), likely reflecting the poor overall gonad development of these oysters.

Testis weight, sperm production, and fresh sperm motility were significantly positively correlated (*p* < 0.001, Table 3), indicating gonad development is critical for obtaining a good quantity and quality of fresh sperm. However, the fresh sperm fertility rate did not show any correlations with oyster phenotypes (shell metrics and body weight) (*p* ≥ 0.188), fresh sperm production, or motility (*p* ≥ 0.260).

PTSM was correlated with the post-thaw sperm concentration (*p* = 0.008) but was not correlated with the post-thaw sperm membrane integrity (*p* = 0.710).

Between fresh and post-thaw sperm, post-thaw motility was significantly correlated with the testis weight (*p* < 0.001), fresh sperm production (*p* < 0.001) and fresh sperm motility (*p* < 0.001), but not fresh sperm fertilization rate (*p* = 0.952) (Table 3). Post-thaw membrane integrity did not correlate with fresh sperm production (*p* = 0.219), fresh sperm motility (*p* = 0.587), or fresh sperm fertility (*p* = 0.141).

## 4. Discussion

In general, a germplasm repository must maintain the same genetic diversity and genetic structure as the source of germplasm, therefore, sample collections need to maintain the same allelic and genotypic frequencies as the original population [30]. Germplasm repositories could be in vivo (e.g., aquarium or research populations in a breeding program) or in vitro (e.g., collections of cryopreserved germplasm) [30]. In vivo germplasm repositories face challenges, such as loss of genetic diversity, inbreeding, genetic adaptations to captivity, and accumulation of deleterious genes, and can become costly (personnel and space). This is in contrast to in vitro germplasm through cryopreservation which can keep samples in their original form indefinitely (cryopreservation), and thus the genetic diversities remain constant even with small populations in which genetic drift must be a constant consideration over generations [31,32].

### 4.1. Strategies for Germplasm Collection for Germplasm Repositories

To establish a germplasm repository, the first thing to clarify is the goal of the repository, such as conservation of genetic biodiversity, endangered species, breeding populations, or commercial populations [30]. A clear goal will greatly benefit the establishment of germplasm collection strategies and of the data management plan. Strategies for sample collection for a germplasm repository could include various approaches, such as survey, exploration, and rescue missions, targeting the capture of the highest possible amount of genetic diversity between and within populations with a minimum number of samples. In the current study, the sperm repository was for preservation of the base populations of a breeding program which intended to create a genetically diverse mosaic by incorporating a total of 102 male and female oysters from 17 sites (six oysters from each site) along the coast in the Gulf of Mexico. All 102 males were included in this sperm repository.

### 4.2. Data Management Plan for Oyster Sperm Repository

For proper use and security of a germplasm repository, a data management plan, including parameters to be collected and managed, definitions and rules for data collection, entry, storage, and sharing need to be in place before sample collection [33]. The FAIR principles (Findable, Accessible, Interoperable, and Re-usable) for scientific data management [34] can be applied for germplasm repositories. For animal [35] and plant germplasm [36], data management plans have been well-developed, and for aquatic animals, the data management plan in zebrafish *Danio rerio* (personal communication with the Zebrafish International Resource Center) has also been well-established. Based on the existing data and goal of this sperm repository in the current study, the data management plan was developed with six categories (Figure 3), including broodstock oyster data (such as species, breed, line, registration number, pedigree information, phenotypic and genomic information) and germplasm (such as viability, number of doses, location in the cryobank) which were digitally recorded.

Germplasm quality analysis is an important aspect for a germplasm repository. A comprehensive review has summarized the current sperm analysis methodology, including new emerging genomic tools [37]. Production of reactive oxygen species (ROS) was considered as a major factor causing sperm cell damage [38], but it is debatable, since cell damage brought by cryopreservation was more severe than those brought by ROS (Yang et al., in review). Considering sperm cell structures and possible impairments during cryopreservation, other sperm quality analyses include: (1) Plasma membrane: membrane integrity, changes in membrane fluidity and components, lipid peroxidation, and protein oxidation; (2) mitochondria: sperm motility and velocity, mitochondrial membrane potential, and ATP release; (3) DNA: chromatin fragmentation, methylation of DNA, cross-linking of DNA, and nitrogen base oxidization; and (4) RNA: oxidation and destabilization. In this study, sperm production, motility, fertility, and membrane integrity were employed for sperm quality evaluation.

Linking genetic data with germplasm stored in a repository is a fundamental component of the data management plan. In a breeding program, genetic information comes from the phenotype and genotype of the male preserved and available information on the genotypes and phenotypes of its ascendants, collaterals, and descendants. It can provide insight into genetic and epigenetic changes [39]. In zebrafish, cryopreservation was found to cause molecular alterations in key genes and transcripts undetectable by traditional assays [40]. Additionally, genotyping data could serve as a relevant output in the upcoming years to reveal whether the natural variability of the cryopreserved populations still maintains its value for the generated offspring and verify that it does not have any genotypic and/or phenotypic destabilizing effects. In plant germplasm cryopreservation, an evolving concept of “cryo-bionomics” was proposed [39] with two study aspects, including the linkage between cryoinjury and stability in vitro and the functionality of plants recovered from cryopreserved germplasms after they were reintroduced into natural environments. Specifically, analysis of genotyping data can detect the genetic variations at each breeding generation.

### 4.3. The Season for Oyster Sperm (Gamete) Collection and Cryopreservation

Gamete collection (quantity and quality) is reliant on the gonad development condition. In this work, the gonad development condition was poor and led to a low quantity and quality of gametes for the repository. Natural reproduction of molluscan bivalves is seasonal and involves gonadal development, sexual maturity, release of gametes (spawning), and re-generation of gonads. The timing of these reproductive stages varies depending on the species and geographical distribution [41]. For Eastern oysters, spawning usually occurs in the spring with increasing temperatures, continuing sporadically through summer, and, in warm-water regions, ending with a minor spawning peak in the fall. Control of reproduction in molluscan bivalves involves a complex of exogenous factors, such as temperature, food, salinity, air-drying, and endogenous factors related to the neuro-endocrine cycles [42]. Temperature is probably the most recognized factor that influences gonad development [43]. Food availability (quantity and quality) is another important factor acting in conjunction with temperature [2]. Regulation of the gonadal development is believed to be controlled by the endogenous sensory receptors on nerve ganglia [44]. Along the coast of the Gulf of Mexico, Eastern oysters have a primary spawn in the spring from March to May, spawn sporadically through summer following by another secondary peak in fall from August to September (about 2–4 weeks) followed by more sporadic spawning in late fall. Under culture conditions, of course, reproductive conditions may be manipulated through ‘conditioning’, relying heavily on phased temperature changes and sufficient food availability.

In this study, conditioning of gonad development was not used, instead allowing the broodstock oysters to ‘ripen’ naturally in the field. However, these oyster samples were collected from different locations in spring and summer 2020, and they could have faced changes in the environmental conditions (such as salinity) following transfer from collection sites to maturation sites and may not have been able to adapt to new conditions and develop their gonads between transfer and spawning. For molluscan bivalves, it is generally believed that regeneration of gonad development needs to experience a sufficient time period, often a seasonal period, for energy accumulation [45,46].

Sexual maturity in oysters (and most bivalves) could be evaluated through visual observation of gonad size or color or biopsy of gonads for gamete observation [47]. The fully developed gonad of Eastern oysters could be over 10–12 mm in thickness accounting for 40% of the total body volume [45], and the gonoducts could be observed visually (Figure 4, fully developed gonad). In the current study, the spawning was performed from 31 August to 4 September 2020 rather than during the peak spring spawning season due to delays imposed by COVID-19 pandemic protocols. Although these oysters were temporarily cultured in the AUSL oyster grow-out site, the gonads were overall in poor condition and maturation status varied widely among oysters from the different collection sites. Additionally, testis weight, sperm production, and fresh sperm motility are all correlated regarding the gonad development condition. In our experience, fresh sperm collected from fully developed males (e.g., the one in Figure 4) usually has over 90% motility and sperm concentration can reach over 2–5 × 10^9^ cells/mL when suspending in seawater at five times the testis weight [24,48]. Therefore, our opinion is that the poor gonad condition limited the success of the cryopreserved sperm.

### 4.4. The Method to Collect Oyster Sperm (Gametes) in Oysters for Germplasm Repository

Spawning behavior in bivalves is significantly influenced by the surrounding water [46], and thermal induction of spawning has proven to be a successful approach [43]. To date, manipulation of temperatures has become a routine practice in commercial hatcheries for accelerating the sexual maturity of broodstock and inducing spawning in many molluscan bivalves [41]. With further understanding of the mechanism for the control of gonad development and spawning, more methods were used to trigger the spawning activity [41,49], and can be summarized as: (1) **physical methods**, such as an increase of temperature or salinity, water flow, and air-drying; (2) **chemical methods**, such as injection of serotonin, hydrogen peroxide, and sex steroids; and (3) **biological methods**, such as the addition of heat-treated sperm or crushed testis or microalgae [50,51]. Regardless of induction methods, successful spawning and gamete collection requires sexually mature broodstock from wild populations or hatchery-produced lines during natural maturation and spawning seasons or after culture in controlled conditions for acceleration of gonad development (usually termed “conditioning broodstock”).

For *Crassostrea* oysters (but not most scallops, clams, or mussel species), oocytes collected from mature gonads by physical dissection (termed “strip spawning”) can be fertilized by stripped sperm, and fertilized eggs can develop normally [52]. Thus, strip spawning has become a routine method for oyster commercial triploid seed production and breeding programs because this method allows convenient gamete collection and mating at an arranged time. However, gametes collected by strip spawning may include immature gametes and yield low fertilization. In this study, gamete collection was conducted by strip spawning. Although sperm was collected, the low sperm production, motility, and fertility may be largely accounted for by the inclusion of immature gametes from the poor condition of gonad development.

### 4.5. Streamlined Procedure for Sperm Sample Collection, Processing, and Cryopreservation

The entire process of sperm collection, processing, and cryopreservation described in this study was performed by three staff members after oysters were opened and male oysters were identified by two other staff. Fertilization tests of fresh sperm were performed by separate crew members focused on oocyte collection and fertilization management for the spawning. Before the spawning event, sample nomenclature and breeding strategies were established. Accordingly, sample straws, beakers for testis and sperm suspension, 50 mL graded centrifugation tubes (for holding filtered sperm), and 1.5 mL microcentrifuge tubes for sperm dilution and hemocyte counting were all labelled prior to the spawning day. In addition, a pre-formatted spreadsheet was prepared for entry of daily data records and calculations. With these streamlined arrangements, a total of up to 30 oysters were processed (with a total of 1200–2400 straws) per day. The most time-consuming step was sperm collection, which included testis stripping, sperm suspension, filtering, concentration determination, and motility estimation. Additionally, since the spawning was performed in a field hatchery, sample cooling was performed using a cost-effective and portable aeration freezing system [53]. This home-made system consists of a styrofoam box as a cooling chamber and an aeration system to control the liquid vapor temperature, and can cool 40 or 100 straws per cooling cycle in 10–15 min. A smooth workflow of loading sperm suspension into straws, sealing sample straws, and cooling sample straws was achieved.

## 5. Conclusions

Overall, this study described a streamlined procedure of oyster sperm collection, processing, and cryopreservation for establishing a sperm repository. This sperm repository included a total of 102 male oysters from 17 collection sites along the Gulf of Mexico coast which served as male founders for a breeding program. The data management plan for the sperm repository was developed, including oyster phenotype, genotype, sperm production, fresh sperm quality, cryopreservation, and post-thaw quality. This work offers some suggested techniques and strategies as a template for constructing more oyster germplasm repositories to serve breeding programs and for the conservation of natural resources.

## Figures and Tables

**Table 1:** Summary of germplasm cryopreservation studies in the eastern oyster *Crassostrea virginica.* In the current study, the protocol by Yang et al. [24] was used for sperm cryopreservation for establishing the sperm repository. Abbreviations: DMSO: dimethyl sulfoxide; NA: not available; LN: liquid nitrogen; PG: propylene glycol; ASW: artificial sea water. HBSS: Hanks’ balanced salt solution.

	Medium	Cryoprotectant	Equilibration	Container	Cooling Rate	Thawing	Fertilization	Post-Thaw Survival	Reference
Sperm	Seawater pH = 7.0	DMSO at 5% and 10%	NA	2-mL ampoule	L °C /min from 0 to −8 °C, at 5.5 °C/min to −25 °C, then LN	21 °C	34 mL post-thaw sperm plus million oocytes in 250 mL	1–5% post-thaw motility and 2% fertilization	[21]
	2.6× HBSS	DMSO 8% with 80 mM glycine, 55 mM NaHCO_3_	20 min at 0 °C	0.25-mL plastic straws	5 °C/min to −5, −20, −40, or −80 °C, then plunged into LN	55–60 °C water bath	0.25-mL sperm to 200–900 eggs in 300 mL seawater	7–91% post-thaw fertility	[22]
	ASW or Ca-free HBSS	0, 5, 10, 15, 20, and 25% (*v/v*) PG with/without 0.25 M sucrose	20 min at 21 °C	5-mL macrotubes	2.5 °C/min to −30 °C, and then plunged LN	70 °C for 15 s, 25 °C until thawing	Diluted in Ca-free HBSS at 1:1	57% post-thaw fertility	[23]
	Ca-free HBSS pH = 7.2	Methanol, DMSO, and PG at 10%	10, 20, 30, 40, 50 and 60 min, mixing at 1:1	0.5-mL of French and CBS™ straw	1, 5, 10, 15, 20, 25, 30, and 40 °C/min from 5 to −80 °C	30, 40, and 50 °C water bath	2-mL post thaw sperm mix with 50-mL oocytes at 10,000/mL	18–95% post-thaw fertilization 10% DMSO	[24]
	Ca-free HBSS, pH = 7.2	10% DMSO	20 min after mixing at 1:1	0.5-mL French straw	15 °C/min from 5 to −80 °C, then to LN	40 °C of water bath 6 s	3–72% fertility; self-fertilization 0–43%	8 selfing families confirmed	[26]
Swimming Larvae	ASW	15% PG with 0.25 M Sucrose	20 min at 21 °C	5-mL macrotubes	2.5 °C/min from 15 to −30 °C, hold for 5 min, then to LN	70 °C for 15 s water bath	NA	4 months old (850 spat)	[25]
	HBSS	5, 10, 15, 20, 25% PG with 0.25 M Sucrose	20 min at 21 °C	5-mL macrotubes	2.5 °C/min from 15 to −30 °C, hold for 5 min, then to LN	70 °C for 15 s water bath	D-larvae	24% D-larvae rate	[23]

**Table 2:** Eastern oyster *Crassostrea virginica* collection location, shell metrics, whole body weight, testis weight, gonadosomatic index (percentage of gonad weight out of the total body weight), total sperm production, and fresh sperm motility (%) used for breeding program and sperm cryopreservation. Significant differences in fresh sperm motility among different collection sites are labeled with different letters (a–e) (*p* < 0.050). **FL**: Florida; **AL**: Alabama; **MS**: Mississippi; **LA**: Louisiana; **TX**: Texas.

State	Location	Abb	Height (mm)	Length (mm)	Width (mm)	Weight (g)	Testis Weight (g)	Gonadosomatic Index (%)	Sperm Volume (mL)	Sperm Concentration (×10^9^ cells/mL)	Sperm Production (×10^10^ cells)	Fresh Sperm Motility (%)
**FL**	Corrigan Reef	CR	93.37 ± 6.57	51.01 ± 3.74	28.63 ± 4.32	93.86 ± 12.11	1.52 ± 0.00	0.75 ± 0.42	9.67 ± 4.92	0.09 ± 0.05	0.10 ± 0.11	11 ± 8 ^e^
	Lone Cabbage Reef	LR	94.43 ± 8.43	51.42 ± 4.75	25.36 ± 1.83	78.58 ± 7.19	1.56 ± 0.41	1.00 ± 0.32	13.33 ± 6.32	0.60 ± 0.36	0.91 ± 0.90	23 ± 11 ^c,d,e^
	Seahorse Key	SK	87.37 ± 5.50	50.72 ± 2.84	30.52 ± 3.07	80.53 ± 9.63	1.50 ± 0.70	0.70 ± 0.27	11.75 ± 4.57	0.27 ± 0.13	0.31 ± 0.17	14 ± 4 ^d,e^
	Oyster Bay	OB	90.26 ± 10.56	56.48 ± 5.74	30.09 ± 5.78	106.30 ± 36.27	3.18 ± 1.57	2.50 ± 0.71	22.42 ± 9.08	0.73 ± 0.49	1.59 ± 1.07	70 ± 20 ^a^
	Alligator Harbor	AH	85.68 ± 11.75	48.38 ± 3.63	29.01 ± 3.69	79.49 ± 25.00	2.33 ± 1.26	2.00 ± 0.71	15.58 ± 6.25	1.24 ± 0.63	1.94 ± 1.09	50 ± 24 ^a,b,c,d^
	Pensacola Bay	PB	80.70 ± 7.33	46.57 ± 7.66	27.43 ± 7.89	72.66 ± 23.28	1.62 ± 0.94	1.17 ± 0.26	12.67 ± 6.05	0.44 ± 0.22	0.47 ± 0.18	21 ± 22 ^d,e^
**AL**	Alonzo Landing	AL	88.41 ± 6.69	54.44 ± 4.58	27.84 ± 4.90	78.82 ± 11.09	1.39 ± 0.44	/	11.67 ± 3.40	1.40 ± 0.17	1.61 ± 0.43	43 ± 29 ^a,b,c,d,e^
	Cedar Point	CP	85.48 ± 9.16	63.28 ± 2.98	34.61 ± 5.65	152.11 ± 33.08	2.69 ± 0.57	3.25 ± 1.33	15.08 ± 2.87	2.01 ± 1.12	3.11 ± 1.83	47 ± 5 ^a,b,c,d,e^
**MS**	Pascagoula	PS	80.33 ± 5.00	53.63 ± 5.10	30.74 ± 5.05	105.95 ± 28.91	3.54 ± 1.71	3.25 ± 0.76	20.00 ± 7.27	1.39 ± 0.50	2.82 ± 1.52	60 ± 27 ^a,b,c^
**LA**	Sister Lake	SL	111.87 ± 13.75	73.40 ± 8.34	42.31 ± 4.80	280.50 ± 112.44	2.09 ± 1.22	1.00 ± 0.45	13.58 ± 8.69	0.33 ± 0.24	0.43 ± 0.34	24 ± 19 ^b,c,d,e^
	Lake Calcasieu	LC	91.96 ± 14.67	67.64 ± 11.01	40.93 ± 6.58	211.38 ± 87.83	2.11 ± 0.86	2.25 ± 0.29	18.08 ± 3.67	0.67 ± 0.13	1.19 ± 0.30	40 ± 0 ^a,b,c,d,e^
**TX**	Lake Sabine	LS	92.51 ± 11.17	60.67 ± 5.45	43.40 ± 10.45	221.75 ± 88.11	1.55 ± 0.81	1.08 ± 0.38	13.08 ± 4.34	0.60 ± 0.25	0.78 ± 0.40	62 ± 13 ^a,b^
	West Galveston	WG	96.31 ± 16.45	62.71 ± 6.23	39.90 ± 7.25	198.19 ± 80.14	2.00 ± 0.61	1.83 ± 0.88	10.67 ± 2.66	0.29 ± 0.14	0.30 ± 0.14	22 ± 12 ^c,d,e^
	East Galveston	EG	83.95 ± 5.86	57.40 ± 5.95	35.13 ± 4.29	152.51 ± 27.26	3.27 ± 1.08	2.75 ± 0.87	22.25 ± 4.12	0.73 ± 0.18	1.60 ± 0.40	73 ± 5 ^a^
	West Matagorda	WM	89.44 ± 6.52	62.36 ± 7.09	41.57 ± 5.08	169.60 ± 28.76	1.75 ± 0.54	1.17 ± 0.26	12.00 ± 3.03	0.21 ± 0.15	0.23 ± 0.15	24 ± 22 ^b,c,d,e^
	East Matagorda	EM	92.30 ± 14.47	52.70 ± 11.46	33.01 ± 4.07	115.33 ± 17.07	1.19 ± 0.32	1.50 ± 0.89	15.50 ± 5.15	0.58 ± 0.44	0.94 ± 0.78	41 ± 34 ^a,b,c,d,e^
	San Antonio	SA	92.02 ± 11.50	66.97 ± 6.13	42.34 ± 2.99	213.17 ± 65.58	2.03 ± 1.55	1.17 ± 0.52	13.00 ± 1.87	0.41 ± 0.14	0.53 ± 0.18	27 ± 16 ^b,c,d,e^
	Mean ± SD		90.11 ± 7.29	57.80 ± 7.57	34.16 ± 6.31	141.21 ± 64.46	2.08 ± 0.71	1.77 ± 0.95	14.44 ± 3.39	0.68 ± 0.52	1.05 ± 0.89	38 ± 20

**Table 3:** The correlation coefficients (R values) (upper panel) and *p-*values (lower panel) among shell metrics (mm), whole body weights (g), testis weight (g), total sperm production (cells), fresh sperm fertility (%), post-thaw sperm motility (%), post-thaw sperm membrane integrity (SMI, %), and post-thaw sperm concentration (cells/mL) of Eastern oysters *Crassostrea virginica* across all sampling locations.

Parameters	Phenotypes				Fresh Sperm Quantity and Quality				Post-Thaw Sperm Viability		
	Height	Length	Width	Weight	Testis Weight	Sperm Production	Motility	Fertilization Rate	Motility	SMI	Sperm Concentration
Height	1										
Length	0.436	1									
Width	0.419	0.687	1								
Weight	0.658	0.814	0.838	1							
Testis weight	0.050	0.111	0.084	0.148	1						
Sperm Production	−0.256	0.005	−0.158	−0.122	0.463	1					
Fresh sperm motility	−0.213	−0.041	−0.017	−0.005	0.405	0.550	1				
Fertilization rate	−0.148	0.081	0.048	0.000	−0.083	0.127	−0.003	1			
Post-thaw sperm motility	−0.177	0.071	0.058	0.057	0.368	0.406	0.446	−0.007	1		
Post-thaw SMI	0.097	0.079	0.126	0.150	−0.013	0.138	0.061	0.165	0.303	1	
Post-thaw sperm concentration	−0.092	0.107	−0.040	0.044	0.186	0.355	0.183	0.066	0.295	−0.042	1
Height	<0.001										
Length	<0.001	<0.001									
Width	<0.001	<0.001	<0.001								
Weight	<0.001	<0.001	<0.001	<0.001							
Testis weight	0.658	0.323	0.455	0.187	<0.001						
Sperm Production	0.021	0.963	0.158	0.279	<0.001	<0.001					
Fresh sperm motility	0.057	0.716	0.880	0.967	<0.001	<0.001	<0.001				
Fertilization rate	0.188	0.474	0.673	0.997	0.460	0.260	0.977	<0.001			
Post-thaw sperm motility	0.115	0.528	0.609	0.613	<0.001	<0.001	<0.001	0.952	<0.001		
Post-thaw SMI	0.390	0.484	0.262	0.180	0.910	0.219	0.587	0.141	0.006	<0.001	
Post-thaw sperm concentration	0.412	0.343	0.724	0.694	0.097	0.001	0.102	0.560	0.008	0.710	<0.001

## Data Availability

The data that support the findings of this study are available from the corresponding author upon request.
